# Increased Susceptibility to Pilocarpine-Induced Status Epilepticus and Reduced Latency in TRPC1/4 Double Knockout Mice

**DOI:** 10.3390/neurolint15040095

**Published:** 2023-12-06

**Authors:** Fang Zheng, Kevin D. Phelan, U Thaung Shwe

**Affiliations:** 1 Department of Pharmacology & Toxicology, College of Medicine, University of Arkansas for Medical Sciences, Little Rock, AR 72205, USA; 2 Department of Neurobiology & Developmental Sciences, College of Medicine, University of Arkansas for Medical Sciences, Little Rock, AR 72205, USA; phelankevind@uams.edu

**Keywords:** TRPC channels, EEG, status epilepticus

## Abstract

Canonical transient receptor potential channels (TRPCs) are a family of calcium-permeable cation channels. Previous studies have shown that heteromeric channels comprising TRPC1 and TRPC4 mediate epileptiform bursting in lateral septal neurons and hippocampal CA1 pyramidal neurons, suggesting that TRPC1/4 channels play a pro-seizure role. In this study, we utilized electroencephalography (EEG) recording and spectral analysis to assess the role of TRPC1/4 channels in the pilocarpine model of status epilepticus (SE). We found that, surprisingly, TRPC1/4 double knockout (DKO) mice exhibited an increased susceptibility to pilocarpine-induced SE. Furthermore, SE latency was also significantly reduced in TRPC1/4 DKO mice. Further studies are needed to reveal the underlying mechanisms of our unexpected results.

## 1. Introduction

Status epilepticus (SE), defined as unremitting or prolonged seizures, afflicts between 50,000 and 150,000 Americans each year [1,2,3,4]. It is the second most common neurological emergency, and is associated with high mortality and high morbidity in adults [1,2,3,4]. Although better treatment guidelines emerged recently [4], SE is still only terminated with drug treatment in a limited group of patients. At least one-third of SE cases are refractory and associated with poor clinical outcomes [5]. Therefore, there is a pressing need to develop new treatment options [5,6]. To achieve this goal, a better understanding of the pathophysiological mechanisms underlying SE is needed.

The long-held belief is that SE occurs when the mechanisms that terminate seizures fail. However, the mechanisms that normally prevent and limit seizures to brief periods remain poorly understood. The progression of SE is stereotypical in patients and in animal models of SE [7,8]: it starts with a “latent” or “foreshadowing” period, followed by a brief period of short bursts of tonic–clonic seizures, a condition which then becomes self-sustaining. Earlier studies revealed a progressive loss of normal GABAergic inhibition [8] and a corresponding increase in NMDA receptor-mediated excitation during self-sustained SE [6]. It remains uncertain whether these changes are sufficient to produce sustained seizures.

Pilocarpine-induced SE is the most widely used animal model of SE [9,10,11]. This model of SE is preferred because it reproduces two hallmarks of human temporal lobe epilepsy: neurodegeneration in the hippocampus and spontaneous recurrent seizures. In this model, pilocarpine, a muscarinic agonist, induces seizure activities in the hippocampus, which subsequently spread to the cortex [9,12,13]. In electroencephalography (EEG) recordings, SE is preceded by a latent period and a transition period, in which bursts of ictal activities are followed by postictal depressions [14,15]. Little is known about the underlying pathophysiological events that establish self-sustained SE.

Recent studies have revealed that canonical transient receptor potential channels (TRPCs) play critical, but temporal and divergent, roles in pilocarpine-induced SE. TRPC channels belong to the transient receptor potential (TRP) super family of cation channels [16,17,18,19,20]. Of the seven TRPC family members (TRPC1-7), a subgroup of closely related TRPC channels (TRPC3, 6, and 7) is consistently pro-convulsive [21,22,23]. The role of the other subgroup of TRPC channels (TRPC1, 4, and 5) in seizures may be more multifaceted. Members of this subgroup can form either homomeric or heteromeric tetrameric channels in artificial expression systems [24,25,26,27,28,29]. The exact subunit composition of native TRPC channels comprising the TRPC1/4/5 subgroup remains controversial. A recent study showed that a large portion of native TRPC channels in the brain were hetero-tetramers, comprising TRPC1, 4, and 5 [30]. However, there is emerging pharmacological and genetic evidence that homomeric TRPC4 or TRPC5 channels exist in the brain and could play distinct functional roles. There is strong evidence supporting a pro-seizure role for heteromeric TRPC1/4 channels. Heteromeric TRPC1/4 channels mediate epileptiform burst firing in both lateral septal neurons and hippocampal CA1 pyramidal neurons [31,32,33]. Since epileptiform bursting has been considered as the cellular equivalent of seizures, one expects that TRPC1/4 double knockout (DKO) mice would show reduced susceptibility to SE. However, we did not detect any reduction in SE susceptibility in TRPC1/4 DKO mice, despite a clear reduction in SE-induced fatality and neuronal cell death [31]. This failure could be caused by the technical limitations of our previous studies, in which we relied on behavioral assessment of SE using the Racine scale [31,32]. This approach is subjective and has severe shortcomings [15].

In recent years, we have used EEG recording to assess the progression of SE and have developed quantitative approaches through which to better characterize pilocarpine-induced SE. These quantitative approaches allowed us to revisit the issue regarding the role of TRPC1/4 channels in SE. In this study, we utilized these approaches to analyze EEG signaling recorded in WT, TRPC1 KO, and TRPC1/4 DKO mice. Our results revealed an increased susceptibility in TRPC1/4 DKO mice, but not in TRPC1 KO mice. Furthermore, we showed that the duration of the latent period and the latency to SE were significantly reduced in TRPC1/4 DKO mice.

## 2. Materials and Methods

*EEG Surgery*: Adult mice (3–5 months old) were used for this study. Mice were anesthetized with isoflurane (4% for induction, and 2–3% for maintenance), and were subsequently mounted in a stereotaxic device, using standard ear bars and a jaw mounting clamp. The skin on top of the head was shaved and swabbed with betadine/alcohol. A small midline incision was made in the skin, and the skin was retracted to expose the skull. A 21-gauge needle was used to place a guide hole into the skull at predefined regions relative to skull sutures, as described previously [15]. Small screws, with wire leads attached, were placed in the guide holes until secure. The leads from 5 screws (4 EEG leads and 1 ground lead) were soldered onto a pin head mount that was secured to the dry skull bone using acrylic dental cement. A topical antibiotic ointment was applied to the skin around the head mount. Mice were placed on a warming pad after surgery and monitored until fully recovered from the anesthesia. After surgery, mice were allowed to recover and adapt to the head mount for 4–7 days.

*EEG/Video Recording*: For recording EEG signals, mice were placed in a round EEG recording cage, with their head mounts attached to a pre-amplifier with a high-pass filter and digitizer (Pinnacle Technology, Inc., Lawrence, KS, USA). The pre-amplifier and cable connected to the digitizer were suspended from above on a swivel arm to allow mice free movement within the round cage, but without enough slack to allow them to chew the wires. A video camera was mounted above the round cage. EEG signals were recorded at a sampling rate of 100 Hz for 7–23 h per recording session. Video signals were recorded at 30 frames/sec for the duration of EEG recording. Mice had free access to food and water during this period.

*Pilocarpine-induced SE*: After baseline EEG signals were recorded for 20–30 min, mice were administered methylscopolamine (10 mg/kg; i.p.), a muscarinic antagonist that does not cross the blood–brain barrier, to block peripheral muscarinic receptors. Pilocarpine, a muscarinic agonist that crosses the blood–brain barrier, was administered later to induce seizures. Convulsive seizures were typically observed within 10 min after the administration of pilocarpine, in a dose-dependent manner.

*Power and Spectral Analyses of EEG signals*: Fast Fourier power spectral analyses of EEG signals were performed as described previously [15,22]. The root mean square (RMS) power was calculated using a rolling 10 sec window using Sirenia Seizure Pro (Pinnacle Technology Inc.). A Hanning window was applied to reduce spectral leakage. The bandwidths for the full, delta, theta, alpha, beta, and gamma frequency bands were set as 0–1000 Hz, 0.5–4 Hz, 4.5–7.5 Hz, 8–13 Hz, 13–30 Hz, and 35–45 Hz, respectively. Ictal activities were determined using an RMS threshold set at 90% of the mean RMS during the SE phase. The latent period was defined as the period between pilocarpine administration and the first ictal activity. The transition period was defined as the period between the first ictal activity and the onset of SE, which was determined via scanning, using the same RMS threshold.

## 3. Results

### 3.1. TRPC1/4 DKO Mice Exhbit Increased SE Susceptibility

In this study, we relied on the pilocarpine model of SE, because it is the only chemical convulsant model that reliably exhibits SE in EEG recordings of mice. In addition to the pilocarpine model of SE, pentylenetetrazol (PTZ) and kainic acid (KA) are also frequently used in the literature to induce SE in mice. However, both failed to elicit sustained and full-spectral seizures in mice (Supplemental Figure S1 and Appendix A).

**Figure 1:**
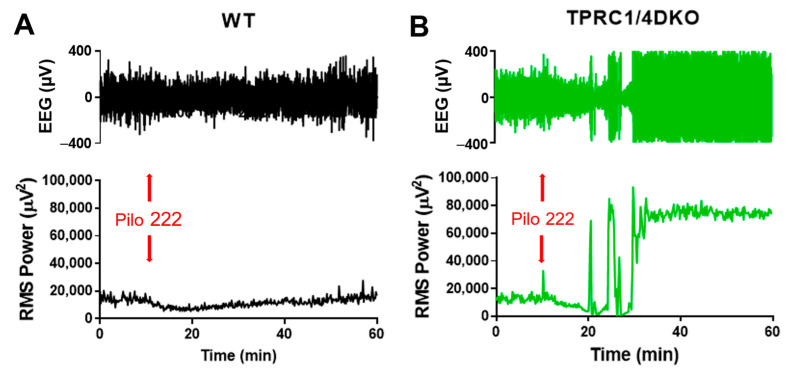
TRPC1/4 DKO mice exhibit increased susceptibility to pilocarpine-induced SE. EEG signals were recorded as described previously [18], and the total RMS power (0.5–1000 Hz) was calculated using a rolling 10 sec window using Sirenia Seizure Pro (Pinnacle Technology Inc.). Mice were pretreated with methylscopolamine (10 mg/kg; i.p.) 20 min before the administration of pilocarpine (Pilo; 222 mg/kg; i.p.; red arrows) to block the peripheral effects of Pilo. This moderate dose of Pilo suppressed EEG activity in 11 out of 12 WT mice (**A**), and in TRPC1 KO mice (n = 4). However, the same dose of Pilo induced SE in 7 out of 7 TRPC1/4 DKO mice (**B**).

The dose–response curve for pilocarpine-induced SE is rather steep in WT mice. A third log scale reduction in pilocarpine dosage, from 280 mg/kg to 222 mg/kg, reduced the incidence of SE from 88% [31] to 8%. There was only a suppression of cortical EEG activity in 11 of 12 WT mice that lacked SE (Figure 1A). Only one WT mouse showed SE at 222 kg/kg pilocarpine dosage. In contrast, all seven TRPC1/4 DKO mice exhibited cortical SE in EEG recordings at 222 mg/kg pilocarpine dosage (Figure 1B).

**Figure 2:**
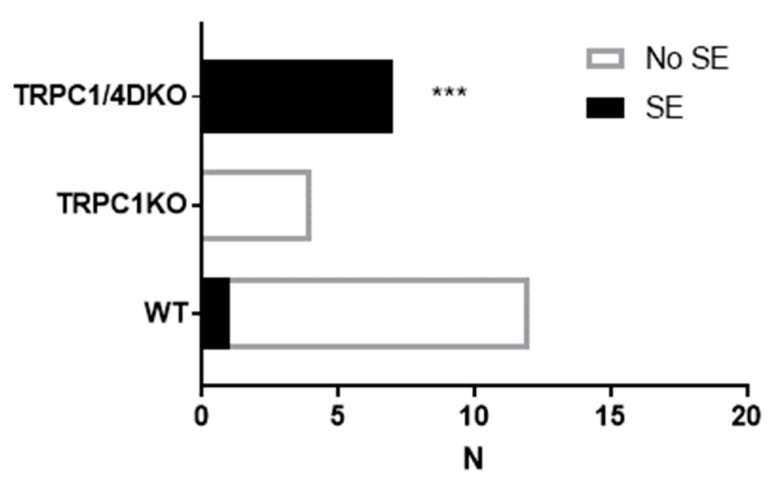
Contingency table analysis of Pilo-induced SE. All mice were administered 222 mg/kg pilocarpine (i.p.). ***: *p* < 0.001, Fisher’s exact test, two-tailed.

In contrast to TRPC1/4 DKO mice, TRPC1 KO mice did not show an increased susceptibility to pilocarpine-induced SE (Figure 2). At 222 mg/kg, pilocarpine induced suppression of EEG activities in all TRPC1KO mice. This difference between TRPC1 KO and TRPC1/4 DKO mice implies that the increased susceptibility to SE in TRPC1/4 DKO mice is not related to heteromeric TRPC1/4 channels. More likely, homomeric TRPC4 channels exert a seizure-impeding influence.

### 3.2. TRPC1/4 DKO Mice Exhibit Early Cortical Seizures and Reduced SE Latency

**Figure 3:**
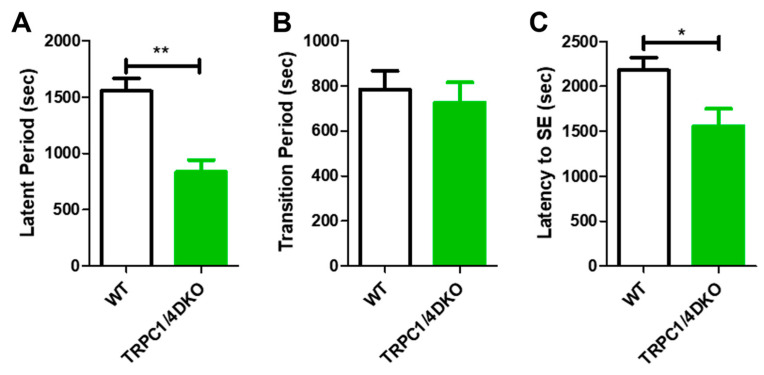
Comparison of latent period (**A**), transition period (**B**), and SE latency (**C**) in WT and TRPC1/4 DKO mice. SE was induced with 280 mg/kg pilocarpine in WT mice (n = 13) and with 222 mg/kg pilocarpine in TRPC1/4 DKO mice (n = 5). (*: *p* < 0.05; **: *p* < 0.01; unpaired *t*-test).

In addition to increased SE susceptibility, we noticed that cortical seizures and SE onset appeared early in TRPC1/4 DKO mice in comparison to WT mice. This prompted a formal comparison of SE progression between the groups, using the quantitative approach described previously [15]. SE progression after the administration of pilocarpine can be divided into three phases: (1) the latent period, characterized by an overall suppression of EEG activity and a late emergence of gamma waves; (2) the transition phase that begins at the emergence of the first cortical seizure and ends with the onset of SE; and (3) the sustained SE phase that last several hours [15]. In TRPC1/4 DKO mice, the duration of the latent period was significantly reduced (Figure 3A), whereas the duration of the transition period was not significantly altered (Figure 3B). Because of the lack of change in the duration of the transition period, the early onset of SE in TRPC1/4 DKO mice (Figure 3C) was primarily caused by the shorter latent period.

### 3.3. Spectral Analysis of SE Progression in WT and TRPC1/4 DKO Mice

**Figure 4:**
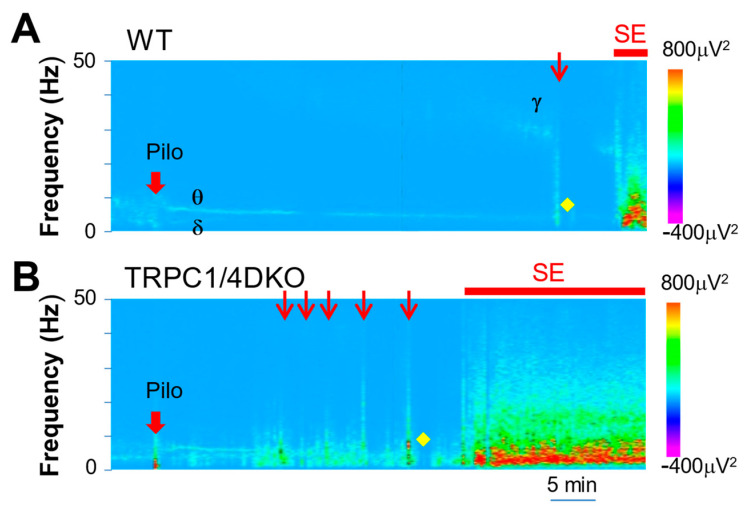
Spectral heat map from a representative WT mouse (**A**) and a TRPC1/4 DKO mouse (**B**). Note that, after the administration of Pilo (280 mg/kg; i.p.) in WT mice, a build-up of gamma waves preceded the first cortical ictal activity (red arrow). In TRPC1/4 DKO mice, bursts of cortical ictal activity suddenly occurred, without preceding gamma waves, after the administration of Pilo (222 mg/kg; i.p). On the other hand, post-ictal depressions (marked with yellow diamonds) were prominent in both WT and TRPC1/4 DKO mice.

To explore the differences between WT and TRPC1/4 DKO mice in critical events leading to SE onset, we performed spectral analysis [22], which revealed detailed patterns of neural activity over time. As reported previously, pilocarpine elicited a synchronization of EEG activity in WT mice, leading to prominent delta and theta waves (Figure 4A). A similar synchronization of EEG activity was also observed in TRPC1/4 DKO mice (Figure 4B). A clear difference between WT mice and TRPC1/4 DKO mice was the presence or absence of gamma waves in relationship to cortical seizures. In WT mice, seizures were always preceded by a buildup of gamma waves (Figure 4A). In TRPC1/4 DKO mice, there was no such buildup, and seizures appeared suddenly (Figure 4B).

**Figure 5:**
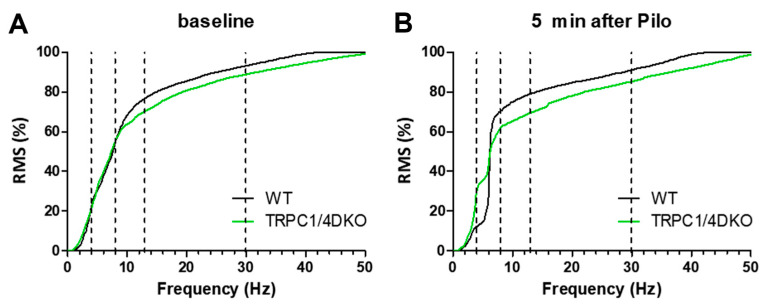
Cumulative distribution curves of WT mice and TRPC1/4 DKO mice. The spectral data from a 4 min window, centered at 5 min before (**A**) and 5 min after (**B**) the administration of Pilocarpine (280 mg/kg for WT mice, 222 mg/kg for TRPC1/4 DKO mice), were analyzed with Sirenia Seizure Pro to derive the spectral data and cumulative distribution curves.

The normalized cumulative distribution curves were very effective in illustrating the subtle differences or changes in EEG activity. We used this approach here to compare the baseline EEG activities and early changes induced with pilocarpine in WT mice and TRPC1/4 DKO mice. In both WT and TRPC1/4 DKO mice, the baseline EEG activities consisted mostly of delta and theta wave, and there was only a very small difference in higher-frequency EEG waves (Figure 5A). After the administration of pilocarpine, strong delta and theta waves emerged in both WT and TRPC1/4 DKO mice (Figure 5B). One difference between WT mice and TRPC1/4 DKO mice was that delta waves were more prominent in TRPC1/4 DKO mice (Figure 5B). Another difference between WT mice and TRPC1/4 DKO mice was that higher-frequency waves, such as beta waves and gamma waves, were also more prominent in TRPC1/4 DKO mice (Figure 5B).

### 3.4. SE intensities Were Comparable in WT and TRPC1/4 DKO Mice

We compared the SE intensities at 60 min after the administration of pilocarpine. As shown in Table 1, the total RMS values in the full frequency range for both WT mice and TRPC1/4 DKO mice were comparable. Detailed comparisons of the delta, theta, alpha, beta and gamma frequency ranges also showed no significant differences between WT and TRPC1/4 DKO mice. Collectively, there was no detectable difference between SE observed in WT mice and SE observed in TRPC1/4 DKO mice.

These observations indicate that the difference between WT mice and TRPC1/4 DKO mice is primarily in the latent period. Once established, SE in TRPC1/4 DKO mice progressed in a similar manner to SE in WT mice.

## 4. Discussion

In this study, we investigated the susceptibility to pilocarpine-induced SE in TRPC1/4 DKO mice using EEG recording and quantitative data analysis. This quantitative approach is needed because our previous studies on TRPC1/4 DKO completely relied on the subjective grading of convulsive behaviors using the Racine scale [31]. Relying purely on subjective grading using the Racine scale to determine whether an animal reaches the state of SE is problematic, and the precise time of onset is often difficult to discern [15]. These difficulties with seizure grading using the Racine scale partially contributed to our failure, in an earlier study [31], to detect significant differences between WT mice and TRPC1/4 DKO mice. The limited availability of TRPC1/4 DKO mice at the time also prevented us from testing the intermediate dosage of pilocarpine (222 mg/kg), which showed the drastic difference between WT and TRPC1/4 DKO mice reported in this study. Our quantitative analysis of EEG data has now revealed an increase in SE incidence in TRPC1/4 DKO mice.

In addition to increased SE susceptibility, TRPC1/4 DKO mice also exhibited a greatly reduced latent period and reduced SE latency. This lengthy latent period is unique to pilocarpine-induced SE, and PTZ and KA produce ictal activities and convulsions within 5 min after the administration of the chemical convulsant (Supplemental Figure S1). The site of action of KA is generally considered to be the KA receptors in the hippocampus [34,35,36]. It elicited convulsive behaviors, such as Straub’s tail and rearing, without full-spectral ictal activities in EEG recordings (Supplemental Figure S1). PTZ, a chemical convulsant that inhibits GABA-A receptors [37], elicited full-spectral ictal activities (Supplemental Figure S1) and a different repertoire of convulsive behaviors [38]. Pilocarpine activates presynaptic muscarinic receptors on the glutamatergic terminals and GABAergic terminals, causing the inhibition of both excitatory and inhibitory neurotransmission. This is clearly shown in the suppression of cortical EEG signals and is associated with “immobility” and “staring”. The convulsive behavior that occurs during the latent period after pilocarpine administration is limited to facial twitching, head nodding, and forelimb clonus (stages 1–3 on the original Racine scale). In other words, the convulsive behaviors rated as Racine Stages 1–3 occur without cortical ictal activities during the silent period after the administration of pilocarpine. We have not performed detailed analysis of video recordings of TRPC1/4 DKO mice during the latent period, due to the limitation of manpower. This is a topic worthy of pursuing further in the future.

Spectral analysis of the latent period revealed no major differences between WT mice and TRPC1/4 DKO mice. After the administration of pilocarpine, increased delta wave and theta wave activities were observed in both WT mice and TRPC1/4 DKO mice. A minor difference is that delta waves dominate in TRPC1/4 DKO mice whereas theta waves dominate in WT mice. Theta waves are considered to be associated with the loss of consciousness, because they are often observed in traumatic brain injury patients or surgery patients undergoing anesthesia [39]. However, delta waves are prominent in conscious patients suffering from Rett syndrome or Lennox–Gastaut syndrome [39]. Therefore, the dominance of delta waves in TRPC1/4 DKO mice could be a contributing factor for the increased SE susceptibility in these mice. Further studies are needed to find out why delta waves become dominant in TRPC1/4 DKO mice and how delta waves are linked to cortical seizures. Another minor difference between WT and TRPC1/4 DKO mice is the noticeable higher-frequency component in the EEG power spectrum after pilocarpine administration in TRPC1/4 DKO mice (Figure 5). The functional impact of this difference is uncertain at the present time. However, it is conceivable that these high-frequency waves induced with pilocarpine could contribute to the increased SE susceptibility and shortened latency observed in TRPC1/4 DKO mice.

Epileptiform bursting has long been regarded as the cellular equivalent of seizures [40]. It has been well established that heteromeric TRPC1/4 channels mediate epileptiform bursting, elicited by the activation of group I metabotropic glutamate receptors in lateral septal neurons in both mice and rats. This conclusion is based on the observations that genetic ablation of either TRPC1 or TRPC4 abolish such epileptiform bursting [31,33]. Epileptiform bursting mediated by heteromeric TRPC1/4 channels was also observed in approximately half of CA1 pyramidal cells in mice [32]. Therefore, it was anticipated that TRPC1/4 DKO would show decreased SE susceptibility. However, our present study revealed that TRPC1/4 DKO mice surprisingly exhibit increased SE susceptibility.

What are the underlying cellular or neural network bases for the increased SE susceptibility observed in TRPC1/4 DKO mice? One possible explanation for the increased SE susceptibility in TRPC1/4 DKO mice is that epileptiform bursting in lateral septal neurons inhibits seizure activity in the hippocampus. The lateral septal neurons are GABAergic interneurons, and are traditionally considered to be involved in the modulation of theta rhythm in the hippocampus [41,42]. It is conceivable that the bursting of lateral septal neurons could inhibit theta activity in the hippocampus by inhibiting cholinergic neurons in the medial septum/diagonal band of Broca area. However, the extent of the lateral septum–medial septum/Diagonal Band of Broca projection has been disputed [43]. Although the direct antiseizure role of the epileptiform bursting of lateral septal neurons could not be ruled out, it is not considered as a likely mechanism for the observed increase in SE susceptibility in TRPC1/4 DKO mice.

Another well-known source of hyperactivity and synchronization in the hippocampus is the CA3 area. CA3 pyramidal cells express high levels of TRPC5, in addition to TRPC1 and TRPC4. The extensive recurrent collaterals (RCs) of CA3 pyramidal neurons provide an anatomical basis for hyperexcitability and synchronization in the CA3 area. Previous studies demonstrated that potentiation of the CA3 RC synapses lead to epileptiform bursting in CA3 pyramidal neurons [44,45]. Conceivably, heteromeric TRPC1/4/5 channels could be recruited at CA3 RC synapses or nearby dendrites to increase the synaptic strength of RC synapses. However, this would likely lead to decreased SE susceptibility, not increased SE susceptibility, in TRPC1/4 DKO mice.

Since heteromeric TRPC1/4/5 channels are strongly connected to epileptiform bursting, that is, the cellular manifestation of seizures, the only plausible hypothesis left is that homomeric TRPC4 channels may exert antiseizure effects. Admittedly, this is a controversial hypothesis because a recent study showed that heteromeric TRPC1/4/5 channels are the most prevalent native channels in the brain, and there is a limited presence of native homomeric TRPC4 channels or TRPC5 channels. However, the lack of change in SE susceptibility in TRPC1 KO mice (Figure 2) provides indirect support for this hypothesis. Clearly, this hypothesis needs to be tested in TRPC4 KO mice in future studies. Selective agonists for homomeric TRPC4 channels would be expected to decrease SE susceptibility. Unfortunately, such drugs have yet to be discovered [46,47,48].

Assuming, indeed, that homomeric TRPC4 channels exist in the hippocampus or other cortical regions of the brain and play an anti-seizure role, there is little hint regarding the possible underlying cellular mechanisms. Homomeric TRPC4 channels and heteromeric TRPC1/4 channels are both calcium-permeable cation channels [25,49,50,51]. Expression of these channels in the soma would lead to both depolarization and increased firing. Therefore, they cannot play opposite roles in SE. To exert distinct functional roles, homomeric TRPC4 channels and heteromeric TRPC1/4 channels need to be expressed at distinctive cellular locations. In the hippocampal CA1 region, strong anti-TRPC4 immunoreactivity was observed not in the pyramidal cell body layer, but throughout all other layers (personal observations). This finding suggests a possible localization of homomeric TRPC4 at numerous synapses along the apical and basal dendrites of CA1 pyramidal neurons. Future studies on the role of homomeric TRPC channels in short-term and long-term synaptic plasticity may reveal the underlying mechanisms that mediate the increased SE susceptibility observed in TRPC1/4 DKO mice.

Regardless of the precise underlying mechanism, the increased SE susceptibility and shortened latency reported in this study provide additional evidence for the emerging notion that homomeric TRPC4 or TRPC5 channels and heteromeric TRPC1/4/5 channels are functionally and pharmacologically distinct. In artificial expressions, homomeric TRPC4 or TRPC5 channels often exhibit double rectifications in their I–V relationship at both negative and positive holding potentials [50], whereas heteromeric TRPC1/4 or TRPC1/5 channels often exhibit a negative region in their I–V relationship around the resting potential of neurons [49]. This negative slop region imparts heteromeric TRPC1/4 or TRPC1/5 channels their ability to mediate epileptiform bursting. Our data from lateral septal neurons suggest that homomeric TRPC4 channels and heteromeric TRPC1/4 channels respond to ML204 and La^3+^ differently, indicating distinct pharmacological properties [52]. There is a degree of urgency to further investigate whether homomeric TRPC4 or TRPC5 channels are functionally and pharmacologically distinct from heteromeric channels comprising TRPC1, 4, and 5. The TRPC1/4/5 subgroup of TRPC channels has been molecular targets for intense drug development research in recent years. Answering this question, regarding the difference between homomeric channels and heteromeric groups comprising members of this subgroup will have significant implications on future drug development.

## Figures and Tables

**Table 1:** The RMS values of SE in WT and TRPC1/4 DKO mice.

Frequency Range	WT (n = 5)	TRPC1/4 DKO (n = 6)
Full (0–1000 Hz)	917,781.1 ± 225,984.3	966,520.4 ± 231,360.0
Delta	71,909.6 ± 7527.5	69,378.0 ± 23,807.7
Theta	62,473.5 ± 14,462.0	62,483.5 ± 16,099.3
Alpha	90,492.3 ± 20,899.5	100,915.5 ± 27,197.3
Beta	181,098.8 ± 42,418.7	202,542.0 ± 59,527.1
Gamma	58,089.1 ± 14,935.9	70,425.1 ± 21,369.4

RMS values are presented as mean ± SD (µV^2^). SE was induced in WT mice with 280 mg/kg pilocarpine (i.p.), and in TRPC1/4 DKO mice with 222 mg/kg pilocarpine (i.p.). An unpaired *t*-test was used for statistical comparison between WT and TRPC1/4 DKO mice (*p* > 0.05).

## Data Availability

Data supporting reported results can be accessed by sending a request to the corresponding author directly.

## Supplementary Materials

The following supporting information can be downloaded at: https://www.mdpi.com/article/10.3390/neurolint15040095/s1, Figure S1: EEG and spectral heatmap of PTZ- and KA-induced seizures.

## Appendix A

In addition to the pilocarpine model of SE, PTZ and KA are also frequently used in the literature to induce SE in mice. However, as shown in Figure S1, the seizures induced with PTZ (75 mg/kg; i.p.) or KA (20 mg/kg; s.c.) cannot be characterized as SE for several reasons. Seizures induced with PTZ were full cortical seizures with comparable intensity to Pilocarpine-induced SE. However, the PTZ-induced seizures were brief and not sustained (Figure S1A). The seizures induced with KA never reached an intensity that is comparable to Pilocarpine-induced SE. Furthermore, they are often brief high-frequency EEG activities that lack clear onset or termination (Figure S1B). For these reasons, neither PTZ-induced seizures nor KA-induced seizures should be considered as bona fide SE.
