# Supplementary figures and images for: Increased Susceptibility to Pilocarpine-Induced Status Epilepticus and Reduced Latency in TRPC1/4 Double Knockout Mice
Source: Neurol Int. 2023 Dec 6;15(4):1469–79. doi: 10.3390/neurolint15040095 (PMC10745782; doi:10.3390/neurolint15040095)

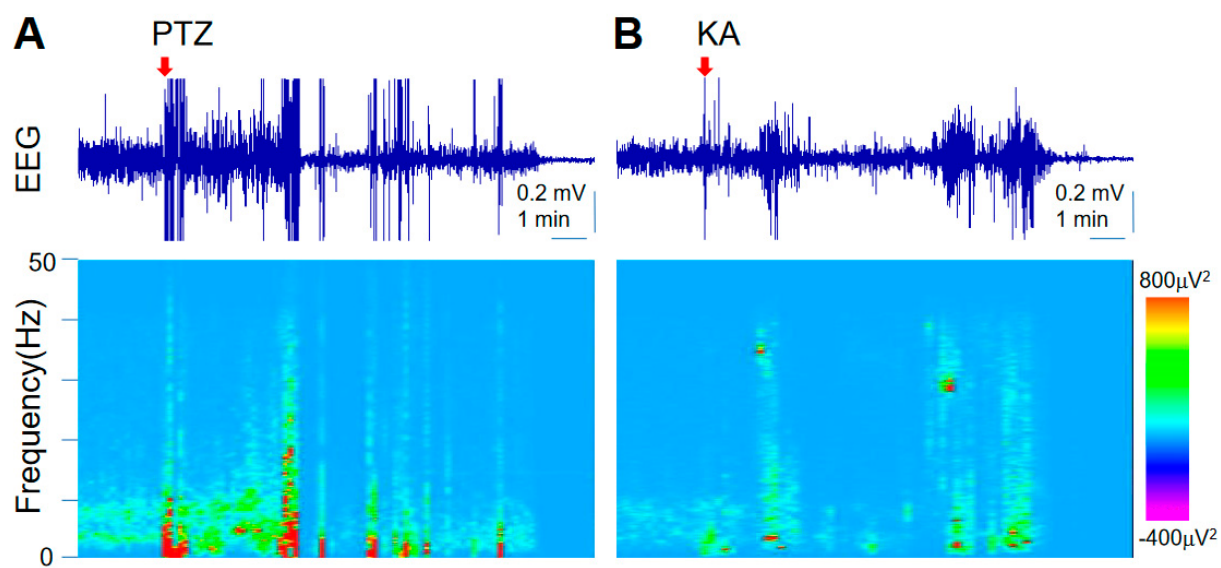

**Figure S1:** EEG and spectral heatmap of PTZ- and KA-induced seizures

Supplement: Supplementary file 1 [file neurolint-15-00095-s001.zip › neurolint-2744439-supplementary.pdf]
